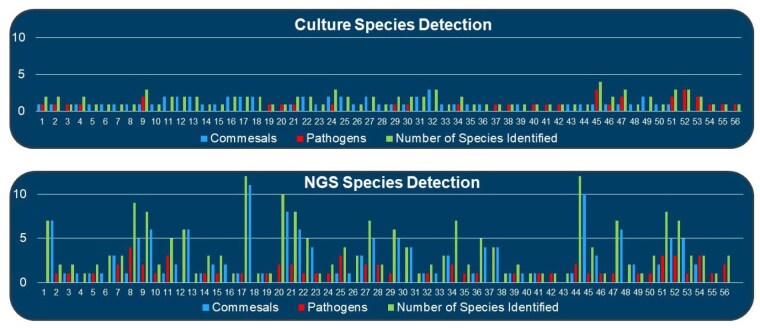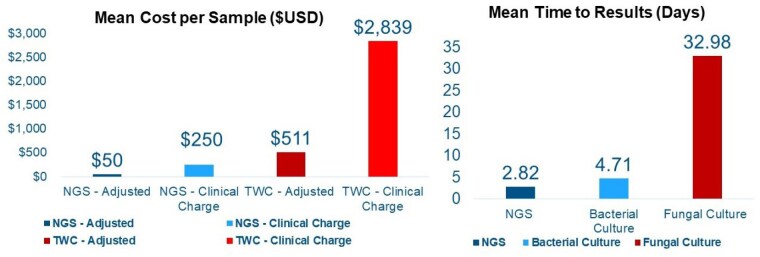# 507 A Pilot Trial Comparing Next-Generation Sequencing and Traditional Wound Cultures Utilizing the Burn Wound

**DOI:** 10.1093/jbcr/iraf019.136

**Published:** 2025-04-01

**Authors:** Deepak Ozhathil, Caroline Corley, Mindy Engevik, Henry Ross, Carter Powell, Lisa Steed, Arman Kilic, Michael Schmidt, Steven Kahn

**Affiliations:** Akron Children’s Hospital; Medical University of South Carolina; Medical University of South Carolina; Medical University of South Carolina; Medical University of South Carolina; Medical University of South Carolina; Medical University of South Carolina; Medical University of South Carolina; Medical University of South Carolina

## Abstract

**Introduction:**

Burn wound colonization is associated with skin graft loss and delayed wound healing. In addition, wound infections are the primary cause of sepsis and death. Unfortunately, traditional wound culture methods (TWC) have afforded little insight about the microbial makeup of burn wounds and historical analysis is lacking. Next Generation Sequencing (NGS) technology represents a promising alternative to TWC, and though validated in other areas of medicine, it has not yet been applied to burn care. In this study we hypothesize that NGS is not inferior to TWC as an efficient and cost-effective modality to characterize the microbiota of burn wounds.

**Methods:**

The study enrolled adults admitted for acute care of burn wounds. A surface wound swab of biofilm and two adjacent tissue specimens were collected from 73 wounds across 38 patients. The swab and one tissue specimen were sent for 16S and 18S amplicon sequencing and metagenomic drug resistance genotyping, while the remaining specimen underwent TWC for microbial speciation and drug sensitivity. Outcomes were assessed with Chi-square and t-tests.

**Results:**

Enrollment favored males (3.8x) with a mean age of 44.5 years. Samples were collected 11.8 days post-injury with a mean surface area of injury of 14.5%. TWC identified one species per sample while NGS identified 3.7 on average. Of the 76 samples collected, NGS detected the same or more species in 55, while 21 samples exhibited discrepancies. NGS had positive results in 9 samples in the setting of no culture growth, while 12 samples identified microbes on TWC that were undetected by NGS. This demonstrated a non-inferiority of 89.5% for bacterial and 81.5% for fungal NGS samples. NGS identified pathogenic bacterial species in 40 samples vs 26 samples by TWC. Days till NGS results for bacteria were 2.81 days compared to 4.65 days for TWC. In contrast, fungal cultures resulted 31.9 days later than NGS. Lastly, billable cost per sample was reduced by a factor of 91.2% for NGS relative to TWC ($250 vs $2,839).

**Conclusions:**

Our data supports that NGS is not inferior to TWC. NGS offers robust and earlier detection of microbial species at a reduced cost per sample. Microbial profiles closer to the date of injury were predominantly commensal/environmental species, while later specimens revealed enteric/pathogenic incursions. Overall pathogen detection occurred at a similar rate between NGS and TWC and a large number of non-pathogenic species were only detected on NGS which is of unclear significance. In conclusion, this study supports the use of NGS as a fast and cost effective alternative to TWC with burn wounds.

**Applicability of Research to Practice:**

NGS has the potential to change how burn care is practiced as it provides information of wound infections much faster than traditional cultures, this is especially important for fungal infections that incur significant morbidity. The wealth of information from this technology also opens the door for the development of new biotherapeutics.

**Funding for the Study:**

Foundation Funding and Institutional Funding